# Supplementary figures and images for: Land consolidation drives changes in soil bacterial community structure and promotes positive bacterial interactions
Source: PLoS One. 2025 Oct 16;20(10):e0334517. doi: 10.1371/journal.pone.0334517 (PMC12530541; doi:10.1371/journal.pone.0334517)

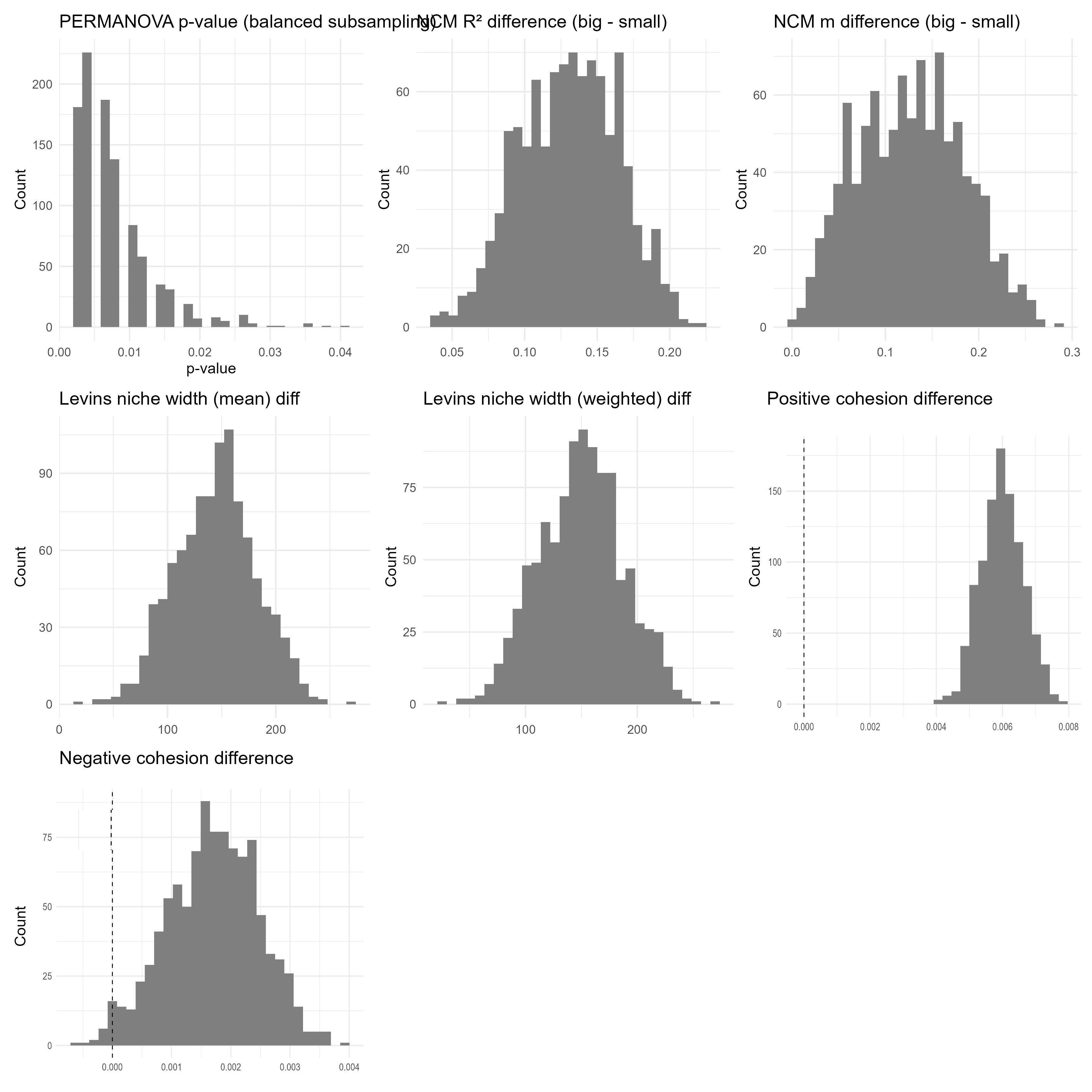

Supplement: S1 Fig — (TIF) [file pone.0334517.s001.tif]

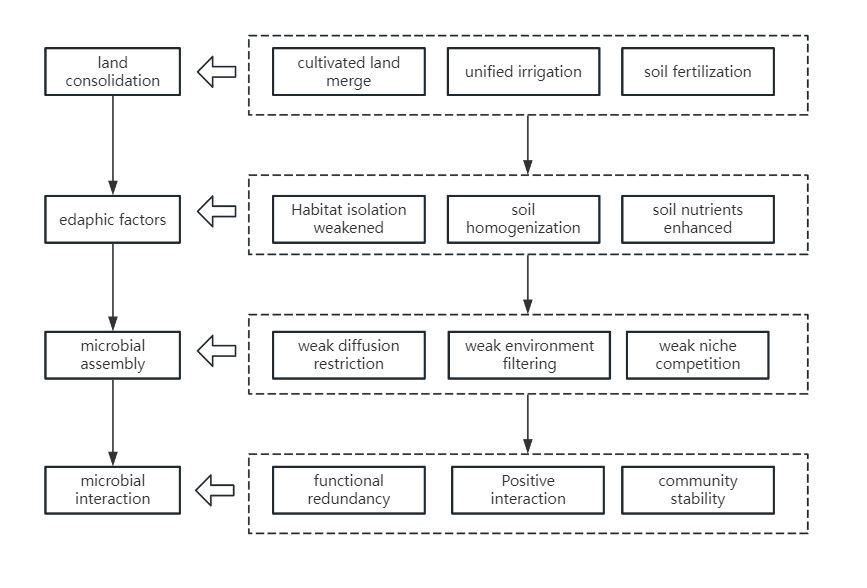

Supplement: S2 Fig — (TIF) [file pone.0334517.s002.tif]

(a)

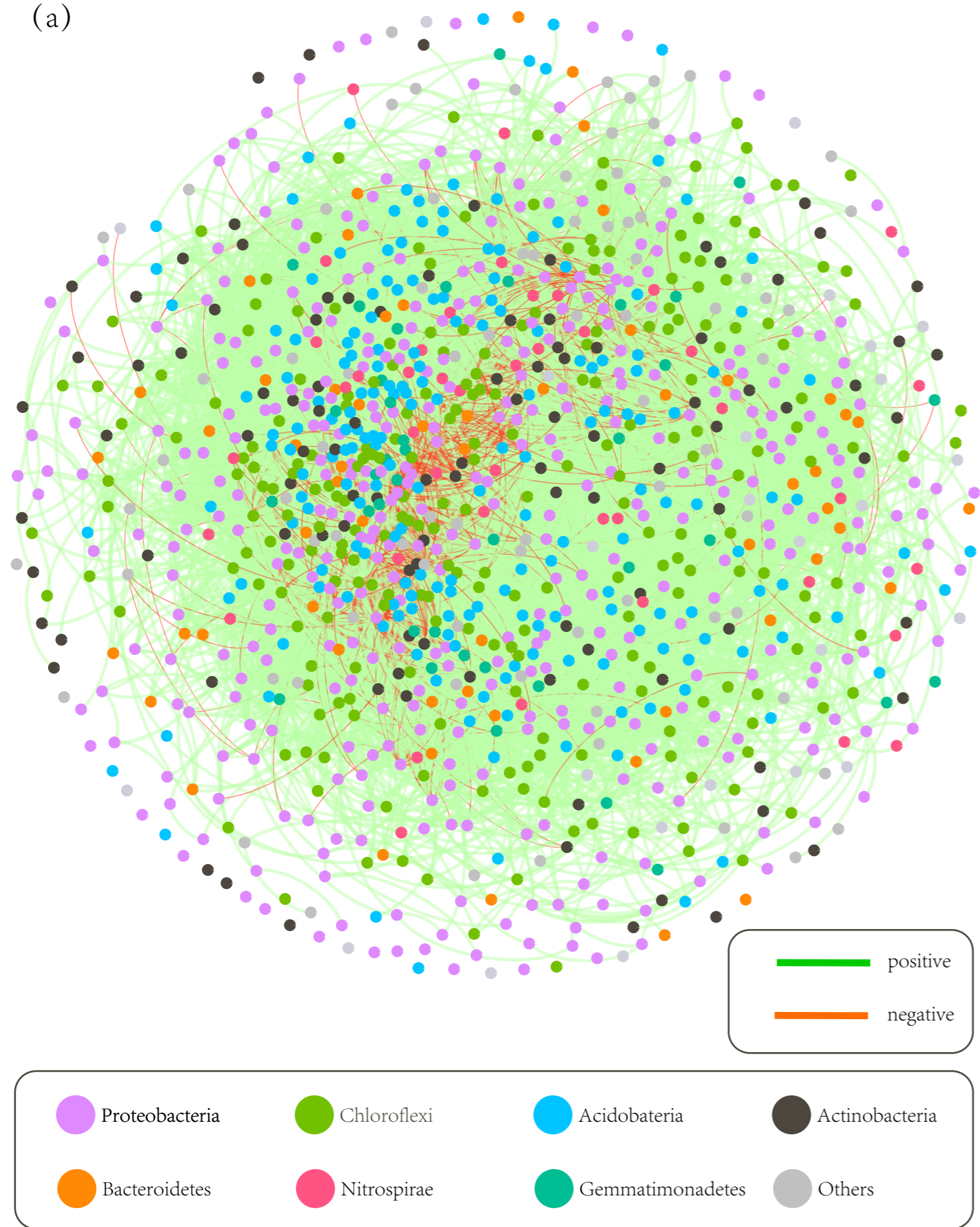

(b)

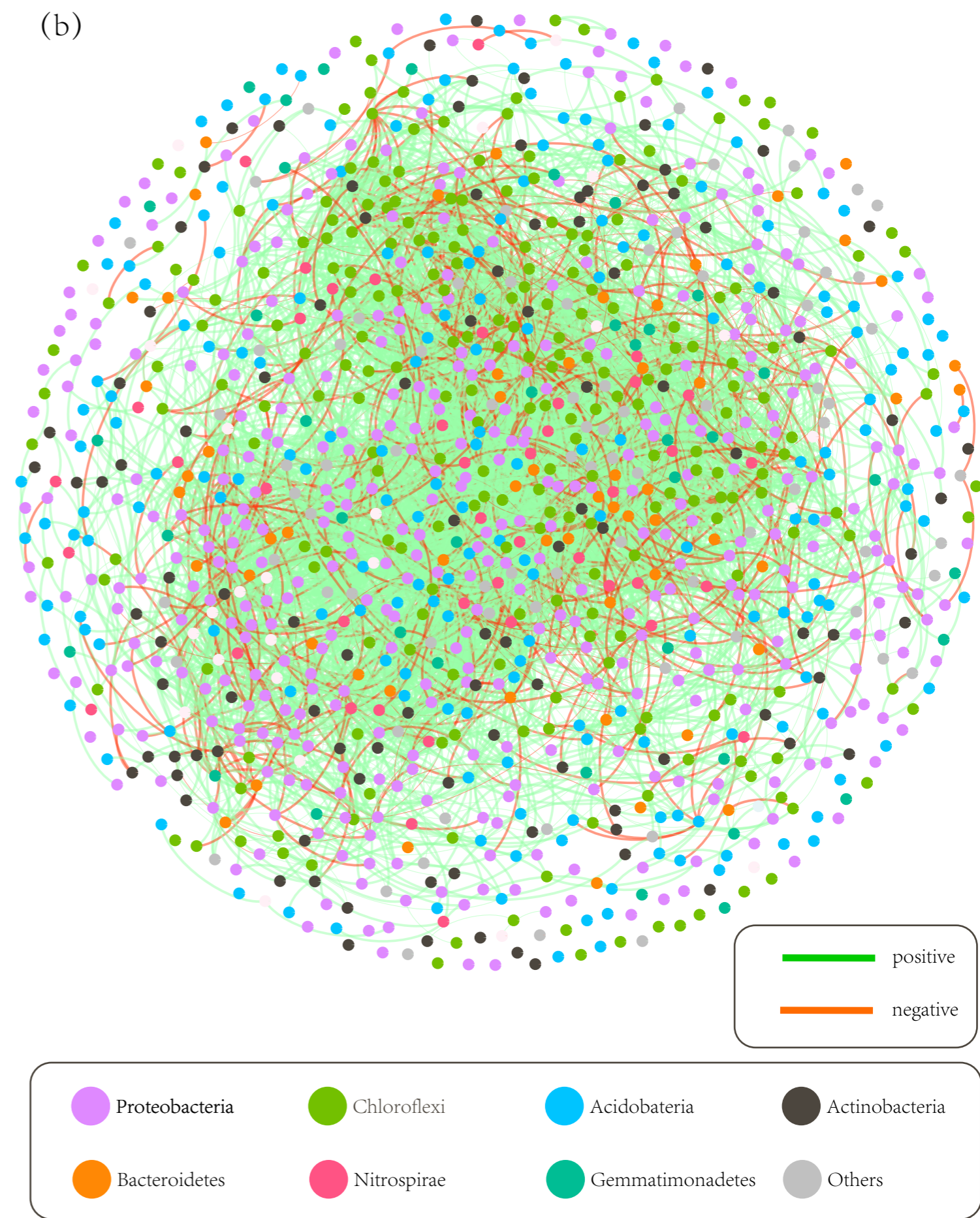

(c)

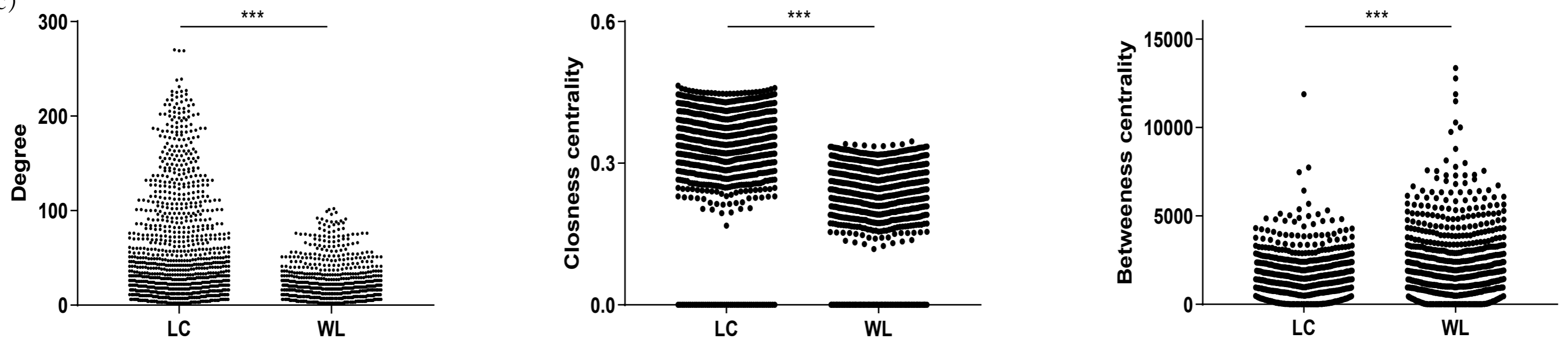

Supplement: S3 Fig — (PDF) [file pone.0334517.s003.pdf]
