# Supplementary material for: Land consolidation drives changes in soil bacterial community structure and promotes positive bacterial interactions
Source: PLoS One. 2025 Oct 16;20(10):e0334517. doi: 10.1371/journal.pone.0334517 (PMC12530541; doi:10.1371/journal.pone.0334517)
Supplement: Attachment — Submitted filename: Response to Reviewers.docx [file pone.0334517.s009.docx]

**Response to Reviewers**

**Manuscript ID:** PONE-D-25-26012
**Title:** Land consolidation drives changes in soil bacterial community structure and promotes positive bacterial interactions

Dear Editor and Reviewers,

We sincerely thank you for your careful evaluation of our manuscript and for the constructive comments and suggestions. We have revised the manuscript thoroughly in response to the comments. Below, we provide a detailed point-by-point response. All changes are marked in the revised manuscript with *Track Changes*.

We have made revisions based on the suggestions of the editor and two reviewers as much as possible, but due to time constraints and existing experimental data, some issues (such as time series sampling, imbalanced sample design, etc.) may only be resolved in future research. It must be acknowledged that the revision is not perfect due to insufficient mastery of some related methods. We would like to thank the reviewers for their feedback. This manuscript has made significant improvements, especially in terms of the rigor of data analysis.

To Editor(Erika Kothe Academic Editor):

**1. In your Methods section, please provide additional information regarding the permits you obtained for the work. Please ensure you have included the full name of the authority that approved the field site access and, if no permits were required, a brief statement explaining why.**

**Response:**

We thank the reviewer for this comment. In the revised Methods section, we have added a statement clarifying that the soil sampling was supported by Jiashan Bureau of Natural Resources (formerly Jiashan County Bureau of Land and Resources). Specifically, no special permits were required for soil sampling as the work was conducted under the framework of the project “Comprehensive Land Consolidation Planning of Jiashan County” organized by Land academy for National Development Zhejiang University. Field access and soil collection were carried out with the consent of local farmers and under the coordination of the Jiashan Bureau of Natural Resources.

**2. Thank you for stating the following financial disclosure:**

**The National Natural Science Foundation of China (No. 42301230) awarded to Y.L, and Zhejiang Provincial Philosophy and Social Science Planning (No. 23NDJ373YB) awarded to L.W.**

**Please state what role the funders took in the study. If the funders had no role, please state: "The funders had no role in study design, data collection and analysis, decision to publish, or preparation of the manuscript."**

**If this statement is not correct you must amend it as needed.**

**Please include this amended Role of Funder statement in your cover letter; we will change the online submission form on your behalf.**

**Response:**

Yao-Ben Lin contributed to the preliminary experimental design and field sampling. Lei Wang contributed to the revision of the manuscript. Both Yao-Ben Lin and Lei Wang provided financial support for the project.

We need to make an additional statement: for the convenience of handling revisions, the current submission lists my email address as the corresponding author’s contact. However, we respectfully request that, upon acceptance, the corresponding author be officially designated as Prof. Lei Wang (email: wanglei@ywicc.edu.cn) in the final publication record. Prof. Wang is the principal investigator of this project and has overseen the overall study design, funding acquisition, and manuscript development. All co-authors have agreed to this designation. Thank you for your understanding.

**3. Please note that your Data Availability Statement is currently missing the direct link to access each database. If your manuscript is accepted for publication, you will be asked to provide these details on a very short timeline. We therefore suggest that you provide this information now, though we will not hold up the peer review process if you are unable.**

**Response:**

The raw sequencing data supporting the findings of this study have been deposited in the NCBI SRA (Sequence Read Archive) under the accession number PRJNA1302308, and are openly accessible at the following URL: <https://www>.ncbi.nlm.nih.gov/sra/PRJNA1302308.

**To reviewer #1：**

**1.Lines 8-10. Only the background sentence is present; methods, key findings and conclusions are missing. Please expand to a structured abstract (Background-Methods-Results-Conclusions) within ~300 words as per PLOS ONE guidelines.**

**Response:**

We thank the reviewer for pointing out this issue. Following the reviewer’s suggestion and PLOS ONE guidelines, we have completely revised the abstract into a structured format (in *abstract*).

**2.Lines 11-15. The Introduction ends without explicit hypotheses or research questions. Please articulate 2-3 testable hypotheses to strengthen the narrative.**

**Response:**

In the revised manuscript, we have added three explicit hypotheses at the end of the Introduction to provide a clearer framework for the study (line 97-102). The new text reads as follows:

“Based on these objectives, we proposed the following hypotheses:

(1): Land consolidation significantly alters soil bacterial community structure.

(2): Land consolidation drives shifts in bacterial community assembly mechanisms.

(3): Land consolidation enhances positive bacterial interactions and network cohesion, thereby promoting greater microbial community stability.”

**3.Lines 18-20. Only the 20-40 cm layer was taken, omitting the typical topsoil (0–20 cm). Provide ecological justification, clarify composite vs. replicate cores, and state whether field blanks were included.**

**Response:**

The corresponding details regarding composite sampling and the inclusion of field blanks have now been added to the *2.1. Experimental design and soil sampling* (line 122-123 and 130-132) section of the Methods. The following is a more detailed explanation about the three questions:

(i) In this study, we focused on the 20–40 cm soil layer, because it is less affected by short-term surface management (tillage, fertilization, vegetation cover) and better reflects the long-term impacts of land consolidation on soil structure and bacterial communities（Eilers, K.，Debenport, S.*et al.*，2012）. Topsoil (0–20 cm) is highly dynamic, but since the land consolidation project has been implemented for over three years, we are more interested in evaluating the relatively stable bacterial changes in the subsoil. Furthermore, this subsurface horizon shows distinct ecological responses and bacterial community differences compared with the topsoil. Several studies have demonstrated that microbial diversity, composition, and functional potential at 20–40 cm are not simply extensions of the topsoil but reflect unique ecological processes and nutrient dynamics （Xu, J.，Song, F.*et al.*，2024;Dong, J.，Wang, P.*et al.*，2025）. Investigating this layer therefore provides valuable insights into the role of subsurface soils in agroecosystem functioning and highlights its importance in shaping bacterial interaction networks under land consolidation practices.

(ii) At each site, soil was collected using a five-point method: one core from each of the four corners of the plot and one from the center. These five cores were homogenized to form a single composite sample for DNA extraction and edaphic analysis. This approach reduces small-scale heterogeneity and ensures that each sample represents the field condition at the plot level.

(iii) We also included field blanks to monitor potential contamination. No substantial contaminant sequences were detected after quality control, and these controls were excluded from downstream analyses.

**4.Lines 63-65. No mention of negative controls, extraction/PCR blanks, or read-quality thresholds. Detail QC pipeline and supply NCBI SRA (or equivalent) accession numbers in the Data Availability statement.**
**Response:**

In the revised Methods section, we have now provided detailed information on the quality-control procedures (line 144-145 and 149-157). Specifically, we included extraction blanks and PCR no-template controls during library preparation; no substantial contaminant sequences were detected. Raw FASTQ files were de-multiplexed with an in-house Perl script, quality-filtered using fastp v0.19.6, and merged by FLASH under the following criteria: (i) reads with an average quality score < 20 over a 50 bp sliding window or length <50 bp after trimming, as well as reads containing ambiguous bases, were discarded; (ii) only overlapping sequences >10 bp with ≤0.2 mismatch ratio were assembled; (iii) samples were distinguished according to the barcode (exact matching) and primers (≤2 nucleotide mismatch). Finally, we have added the data sets were deposited on the NCBI Sequence Read Archive under accession numbers PRJNA1302308.

**5.NMDS and network analyses lack distance metrics, correlation thresholds and FDR correction approach; PCNM/VPA model F and p values are also omitted. Please provide these parameters in Methods and Results.**

**Response:**

(i) We thank the reviewer for this constructive comment. In response, we have added the missing methodological details. Specifically, we clarified that NMDS analyses were based on Bray–Curtis distance (line 161) and reported stress values in the Results (line187).

(ii) For co-occurrence network analysis, in this study, we initially employed Spearman’s correlation with conventional significance testing to construct bacterial co-occurrence networks, which provided an intuitive overview of potential associations among taxa. However, such correlation-based approaches are known to be sensitive to compositionality and multiple testing issues, and thus may overestimate the number of spurious edges. To address this concern, we conducted a new network analysis using a permutation-based null model combined with Benjamini–Hochberg FDR correction. We set the significance threshold at FDR-adjusted *p* < 0.1 to balance statistical stringency with biological interpretability. In microbial co-occurrence networks, the large number of simultaneous comparisons can make overly conservative cutoffs (FDR < 0.05) too restrictive, potentially excluding ecologically meaningful associations. By adopting a more relaxed yet commonly accepted threshold, we ensured that the retained edges were statistically reliable while preserving sufficient network complexity to capture the underlying ecological interactions.

To assess the statistical significance of inferred associations, we implemented a permutation-based null model with 4,000 randomizations to generate empirical p-values for each potential edge. Multiple testing was controlled by applying the Benjamini–Hochberg procedure, and only associations with false discovery rate (FDR) adjusted *p* < 0.10 were retained as significant edges. The convergence of results across both correlation-based and null-model-based frameworks supports the robustness of our findings. And we have made corresponding modifications in the *methods* (section 2.3., line 176-180), *conclusions* (section 3.2.2., line 255-266), and *discussion* (section 4.3., line 378-399).

(iii) For PCNM and VPA analyses, we have added F and p values from permutation tests (999 permutations) to the *Results* section (line 220-222). These details have been incorporated into the *Methods* (line 170-171).

**6.Section 3.2.2 is present but 3.2.1 is missing. Verify section numbering and ensure all figures are cited in order at ≥300 dpi.**
**Response:**

We have carefully re-checked the manuscript and corrected the inconsistencies. Specifically, we revised the section numbering, ensured that all figures are cited sequentially in the text, and updated the figure files to a resolution of ≥300 dpi. These modifications have been incorporated to fully meet the journal’s formatting requirements.

**7.Several citations lack DOIs and journal titles are inconsistently abbreviated. Please format all references per PLOS ONE style (full titles + DOI) and remove extra indents.**
**Response:**

In the revised manuscript, we have reformatted all references according to the PLOS ONE style, ensuring the use of full journal titles, consistent formatting, and the inclusion of DOIs where available (see *references* for details).

**8.To help readers visualize the sampling layout and surrounding context, please add a study-area location map (~1:50,000 scale) with coordinates, and cite it in the “2.1 Study area” or Methods section.**

**Response:**

We appreciate the reviewer’s helpful suggestion. In response, we have prepared a study-area location map showing the geographic coordinates of Jiashan County and the distribution of sampling sites. This new figure has been added as Figure 1 in the revised manuscript and is cited in Section 2.1. *Experimental design and soil sampling*.

To reviewer #2:

**Reviewer #2:** 
**1. The manuscript does not adequately position itself within the broader context of literature on agricultural intensification versus ecological restoration. It is therefore necessary to clarify how consolidation differs from conventional practices such as tillage or fertilization.**

**Response:**

We appreciate the reviewer’s valuable comment. In the revised manuscript, we have added corresponding descriptions in the Introduction. Specifically, we now highlight how land consolidation differs from conventional practices such as tillage and fertilization in line 43-51.

**2.The experimental design includes sufficient spatial replication; however, the comparison of 36 versus 14 sites is substantially unbalanced. A power analysis (e.g., conducted using G*Power or the pwr package) indicates that to detect a medium effect size (Cohen’s d = 0.5) at a significance level of α = 0.05 with 80% statistical power, a balanced sample size of approximately 26–30 per group is required. Such imbalance in the current design may increase the risk of Type II error and potentially lead to inflated effect size estimates.**

**Response:**

We fully acknowledge the reviewer’s concern that our sampling design was unbalanced (36 LC vs. 14 WL). This limitation was indeed present. The reason for this imbalance lies in the actual field conditions: during our field survey, the distribution of consolidated and non-consolidated paddy plots in the study area was highly uneven, as land consolidation had already been widely implemented locally. Our grouping therefore reflected the real situation of land management rather than an ideal balanced design.

We carefully evaluated whether the imbalance might bias our results. We used PERMANOVA (Bray–Curtis) combined with betadisper to ensure that differences were not simply driven by heterogeneous within-group dispersions. Indeed, the PERMDISP test indicated a significant difference in within-group dispersions (F = 4.19, *p* = 0.037), suggesting that group variance heterogeneity may have influenced the PERMANOVA results. To address this issue, we conducted balanced subsampling (repeatedly resampling the larger group to match the smaller group across 999 iterations) and re-ran the PERMANOVA as well as complementary analyses, including the neutral community model, niche breadth, and cohesion metrics. The subsampling consistently showed that the main patterns remained robust (Figure S1), and the specific sub sampling results can be found in the attached file (Table S3.csv). The figure shows the results of balanced subsampling, in which the larger group (LC) was randomly resampled to match the sample size of the smaller group (WL). Histograms represent the distributions of key statistics across iterations: (i) PERMANOVA p-value: Distribution of significance levels for community composition differences. (ii) NCM R² difference and NCM m difference: Differences in the fit of the neutral community model (R² and migration parameter m) between groups. (iii) Levins niche width difference: Differences in ecological niche breadth. (iv) Positive and negative cohesion differences: Differences in network connectivity, indicating stronger positive cohesion under LC while negative cohesion differences remain inconsistent. The specific sub sampling results can be found in the attached file (Table S3.csv).

We believe that these additional analyses help to alleviate concerns regarding the potential impact of unbalanced sample sizes on our findings. Nevertheless, we have also addressed this issue in the Limitations section, where we provide further discussion and outline plans for improvement.

**3. Single time-point sampling fails to capture the dynamic fluctuations across seasons. Paddy soils experience recurring wet-dry cycles that profoundly shape and regulate microbial community structures. It is therefore recommended to either include a dedicated paragraph addressing this temporal limitation or present preliminary data from multiple seasons to better reflect the ecological variability.**

**Response:**

We greatly appreciate the reviewer’s insightful comment regarding the limitation of single time-point sampling. We fully agree that paddy soils undergo recurring wet–dry cycles across seasons, which are known to profoundly shape microbial community composition and assembly processes. Ideally, a multi-seasonal design would allow a more comprehensive assessment of temporal variability.

In this study, we focused on a single sampling campaign because (i) our primary objective was to disentangle the impacts of land consolidation on microbial community structure under comparable agronomic conditions, and (ii) logistical constraints limited us from repeated seasonal surveys. We acknowledge that this temporal limitation may restrict the generalizability of our findings.

To address this, we have added a dedicated paragraph in the Discussion (4.4 *Limitations and future perspectives*, line 418-427) highlighting this limitation and emphasizing that future work should incorporate multi-seasonal or long-term monitoring to capture dynamic microbial responses to both land consolidation and natural hydrological fluctuations. We believe that acknowledging this limitation and outlining directions for future research will clarify the scope of our conclusions.

**4.Consolidation sites may have undergone concurrent improvements in land leveling, fertilization, and irrigation. In the absence of detailed documentation regarding the specific types of fertilizers used, irrigation schedules, pesticide applications, and tillage frequency, it becomes infeasible to isolate the observed effects as being exclusively attributable to “consolidation.” To enhance the accuracy of such attributions, it is recommended to incorporate data from farmer surveys or official government project records.**

**Response:**

We fully agree that land consolidation is usually accompanied by multiple agronomic improvements, such as land leveling, fertilization, irrigation, and pesticide management, and that it is difficult to completely disentangle the individual contributions of these practices from the overall consolidation effect. However, based on our field investigations and farmer interviews, we can confirm that our grouping reflects differences in fertilizer management: consolidated fields implemented improved soil fertilization practices, whereas non-consolidated fields did not. But we must acknowledge that some factors such as irrigation frequency and pesticide management may have been overlooked. Our study was designed to evaluate the overall ecological impact of consolidation as a comprehensive land management strategy, ignoring the isolated effects of certain agricultural practice. We recognize this limitation, and we are currently conducting supplementary surveys and data collection on irrigation schedules, pesticide applications, and tillage frequency, which will allow us to improve attribution accuracy in future studies.

**5.Soil sampling and bacterial samples were collected from the 20–40 cm depth interval; however, it remains unclear whether the geochemical data correspond precisely to this same soil layer.**

**Response:**

In this study, we focused on the 20–40 cm soil layer, because it is less affected by short-term surface management (tillage, fertilization, vegetation cover) and better reflects the long-term impacts of land consolidation on soil structure and bacterial communities（Eilers, K.，Debenport, S.，2012）. Topsoil (0–20 cm) is highly dynamic, but since the land consolidation project has been implemented for over three years, we are more interested in evaluating the relatively stable bacterial changes in the subsoil. Besides, this subsurface horizon shows distinct ecological responses and bacterial community differences compared with the topsoil. Several studies have demonstrated that microbial diversity, composition, and functional potential at 20–40 cm are not simply extensions of the topsoil but reflect unique ecological processes and nutrient dynamics （Xu, J.，Song, F.，2024;Dong, J.，Wang, P.，2025）. Investigating this layer therefore provides valuable insights into the role of subsurface soils in agroecosystem functioning and highlights its importance in shaping bacterial interaction networks under land consolidation practices.

**6.Redox potential (Eh), dissolved organic carbon, Fe(II)/Fe(III) ratios, and bulk density are known to play a critical role in paddy soils but are not included in the current dataset. The inclusion of these parameters could potentially account for a larger portion of the 84% unexplained variance observed in the variation partitioning analysis (VPA).**

**Response:**

We fully agree that redox potential (Eh), dissolved organic carbon, Fe(II)/Fe(III) ratios, and bulk density are critical determinants of microbial community structure in paddy soils. In addition, other factors such as soil organic pollutants and conductivity are also included. However, referring to some previous studies and considering laboratory conditions, we ultimately chose the current experimental factors. We have now acknowledged this limitation explicitly in the discussion (4.4. *Limitations and future perspectives,* line 418-427), noting that their omission likely contributes to the relatively high proportion (84%) of unexplained variance in the VPA results. While these omissions may have contributed to the unexplained variance, our conclusions regarding community assembly remain valid. In future work, we plan to include these measurements to provide a more comprehensive understanding.

**7.Although concentrations of Cu, Cd, Pb, Cr, Hg, and Zn are presented, no significant differences are observed between the LC and WL treatments. It may therefore be appropriate to relocate this table to the supplementary material to maintain focus on the main findings.**

**Response:**

As suggested, we have relocated the table presenting Cu, Cd, Pb, Cr, Hg, and Zn concentrations to the supplementary material (Table S2), since no significant differences were observed between LC and WL plots.

**8.Only 15% of the variance is explained. The remaining 85% should be addressed and may be attributed to potential factors such as unmeasured edaphic variables, biotic interactions, methodological noise, or inherent stochasticity.**
**Response:**

We acknowledge that only 15% of the variance was explained in our VPA results, leaving approximately 85% unexplained. This is a common phenomenon in soil microbial ecology, as community assembly is often shaped by a complex interplay of factors. The unexplained portion may be attributed to unmeasured edaphic variables (e.g., Eh, dissolved organic carbon, Fe(II)/Fe(III) ratios, organic pollutants), biotic interactions such as microbial competition or facilitation, methodological noise, and especially inherent stochasticity in microbial community assembly, leading to our discussion on stochastic processes (in 4.2., line 329-338). Meanwhile, we have also added a paragraph in the Discussion (in 4.4., line 418-427) to explicitly address this issue and emphasize that future studies incorporating more comprehensive environmental and biotic measurements will likely reduce the unexplained variance.

**9.Report the results of goodness-of-fit tests, such as the χ² test or AIC comparisons with niche-based models. Currently, the higher R² value for LC is interpreted as an indicator of stronger stochasticity; however, it could also suggest lower levels of environmental heterogeneity.**

**Response:**

We thank the reviewer for this valuable suggestion. In response, we have now performed χ² goodness-of-fit tests to evaluate the adequacy of the neutral community model (NCM) fits. The results indicated that although the NCM explained a considerable portion of the variation in OTU occurrence frequencies (R² = 0.742 for all samples, 0.785 for LC, and 0.629 for WL), significant deviations between observed and predicted distributions were detected in all cases (χ² tests, *p* < 0.05).However, although these results suggest that while stochastic processes are important drivers of community assembly, the neutral model does not fully capture community patterns. In revising the manuscript, we have refined our statements regarding the role of stochasticity under land consolidation. Rather than overemphasizing stronger stochastic processes, we now highlight that the NCM results mainly reflect enhanced dispersal potential in LC relative to WL, consistent with the expectation that land consolidation improves soil connectivity and reduces dispersal limitation. The *Methods* (line 172-173) , *Results* (line 236-238 and Figure 5), and *Discussion* (line 327-341) sections have been updated accordingly.

**10. The slopes are shallow (−0.1401 vs. −0.2513), and the R² values are very low (0.0064 and 0.0082). Please indicate whether the slopes remain statistically significant after controlling for environmental variables using partial Mantel tests.**

**Response:**

We conducted partial Mantel tests to evaluate whether the distance–decay relationship remained significant after controlling for environmental variables. The results showed that the relationship became not significant (LC, ρ = 0.067, *p* = 0.052; WL, ρ = 0.041, *p* = 0.218; ALL, ρ = 0.052, *p* = 0.072). These findings indicate that the distance effect is largely confounded or masked by environmental heterogeneity. Indeed, this is consistent with the conclusion of 3.1.3, which suggests that spatial factors may not be significant factors affecting bacterial community structure. We have revised the *Methods* (line 165-167), *Results* (line 205-206) and *Discussion* (line 303-309) to include these details.

**11.Elaborate on the Methods section by incorporating comprehensive details regarding the full factorial design, precise GPS coordinates, and a meticulously crafted agronomic management questionnaire.**
**Response:**

In Section 2.1 of the revised manuscript, we have clarified the sampling design in greater detail. Specifically, the consolidated plots were identified based on official land consolidation project records provided by the local Land and Resources Bureau. Taking into account cropping systems, environmental conditions, and rice growth status, we selected a total of 50 sampling sites. Furthermore, to enhance clarity, we have added a study-area map (Figure 1 in the revised manuscript) to illustrate the spatial distribution of these sites.

**12.Please incorporate a supplementary table that meticulously enumerates all edaphic variables for each site, along with corresponding sequencing depths and rarefaction metrics.**

**Response:**

We have provided Table S1, which records detailed physicochemical property data and sequencing information for each site.

**13.Conduct network analyses anew using an additional null-model approach (e.g.,** **SPIEC-EASI), and present the results with FDR-corrected measures of statistical significance.**

**Response:**

We thank the reviewer for this constructive suggestion. In this study, we initially employed Spearman’s correlation with conventional significance testing to construct bacterial co-occurrence networks, which provided an intuitive overview of potential associations among taxa. However, such correlation-based approaches are known to be sensitive to compositionality and multiple testing issues, and thus may overestimate the number of spurious edges. To address this concern, and following the reviewer’s recommendation, we conducted an additional network analysis using a permutation-based null model combined with Benjamini–Hochberg FDR correction (a new Figure 7 in section 3.2.2.), and implemented a permutation-based null model with 4,000 randomizations to generate empirical *p*-values for each potential edge. This stricter procedure substantially reduced the total number of retained edges, yet the overall patterns remained consistent: networks in land consolidation soils were larger, denser, and more enriched in positive associations compared with those in non-consolidated soils. The convergence of results across both correlation-based and null-model-based frameworks supports the robustness of our findings (Figure S3). And we have made corresponding modifications in the *methods* (section 2.3., line 176-180), *conclusions* (section 3.2.2., line 255-266), and *discussion* (section 4.3., line 378-399).

It should be pointed out that we set the significance threshold at FDR-adjusted *p* < 0.1 to balance statistical stringency with biological interpretability. In microbial co-occurrence networks, the large number of simultaneous comparisons can make overly conservative cutoffs too restrictive, potentially excluding ecologically meaningful associations. By adopting a more relaxed yet commonly accepted threshold, we ensured that the retained edges were statistically reliable while preserving sufficient network complexity to capture the underlying ecological interactions.

**14.Include a dedicated section detailing the limitations of the study, such as the temporal snapshot nature of the data and the imbalance in sample sizes across groups.**

**Response:**

In the revised manuscript, we have added a dedicated subsection *4.4. Limitations and future perspectives* to explicitly acknowledge the key limitations of our study (line 418-427).

**15.Conduct a sensitivity analysis by excluding sites characterized by extreme pH levels or organic matter content, in order to evaluate the robustness of the consolidation signal**

**Response:**

In fact, during the initial stage of data processing, we collected 56 soil samples but excluded 6 sites that exhibited outliers. After this quality control step, 50 representative sites were retained and used for all subsequent analyses. Therefore, the consolidation signal reported in our study already reflects a dataset from which extreme outliers had been removed, ensuring the robustness of our conclusions.

Nevertheless, conducting sensitivity analysis is indispensable for strengthening the persuasiveness of the study. To directly address the reviewer’s concern, we further performed a sensitivity analysis, applied the interquartile range (IQR) rule to identify outliers for the main edaphic variables. The thresholds obtained were shown in Supplementary Table S4.

Based on these criteria, three LC sites (LC33, LC34 due to TN > upper IQR, and LC5 due to AK > upper IQR) were excluded. After removal, the dataset included 33 LC samples and 14 WL samples. We then repeated the key analyses, including niche breadth calculation, NCM fitting and cohesion analysis. As shown in Supplementary Table S5, the results before and after exclusion were highly consistent. These results demonstrate that the consolidation signal is robust and not driven by a few outliers.

**16.Meticulous proofreading for grammatical accuracy and typographical errors is essential; furthermore, enlisting the assistance of a professional editing service is highly recommended to enhance overall clarity and linguistic refinement.**

**Response:**

With regard to language, we have carefully proofread the entire manuscript to correct grammatical errors and typographical mistakes. In addition, we plan to seek assistance from a professional English editing service prior to final submission in order to further improve clarity and readability.

**17.Provide a conceptual diagram that succinctly illustrates the sequential process whereby soil consolidation leads to homogenization, fostering a stochastic microbial assembly, which in turn enhances positive cohesion within the soil matrix.**

**Response:**

We have added a conceptual schematic below (Figure S2) in the *Supporting information* to illustrate the sequential mechanism by which land consolidation influences soil microbial communities. As shown in the figure, land consolidation serves as the initial driver, comprising three key measures: (i) cultivated land merging, which reduces fragmentation of small parcels, facilitates mechanized farming, and is often accompanied by land leveling; (ii) unified irrigation, which minimizes local differences in soil moisture through coordinated water management; and (iii) soil fertilization and amendment, whereby consistent applications of fertilizers and conditioners promote chemical uniformity and nutrient enrichment.

These practices collectively lead to soil homogenization and associated ecological effects. Specifically: (i) land consolidation weakens habitat isolation, while unified irrigation provides conditions conducive to microbial migration and dispersal; (ii) homogenization of physical and chemical properties simplifies originally diverse microhabitats, leading to a more uniform soil environment; and (iii) soil nutrient content is enhanced, improving overall fertility.

The homogenized environment exerts strong impacts on microbial community assembly, constituting the microbial response. Land merging and stable moisture supply partially reduce diffusion limitation; habitat simplification directly weakens environmental filtering, diminishing the dominance of microbes adapted to special or extreme environments. As a result, deterministic species sorting becomes less influential, and stochastic processes such as ecological drift and dispersal limitation play a more prominent role, leading to communities characterized by stochastic assembly. Such communities often display greater functional redundancy (different taxa performing similar functions) and reduced niche competition (as resource conflicts decrease under uniform conditions).

The ultimate system-level effects are that functional redundancy and reduced niche competition jointly promote stronger positive microbial interactions (e.g., mutualism and cooperation). The increase in positive associations enhances community cohesion and, through microbial activities such as secretion of extracellular polymers, significantly strengthens the stability of the soil matrix. This sequence highlights that land consolidation not only improves soil physicochemical properties but also reshapes assembly mechanisms and interspecific interactions, ultimately enhancing the stability and resilience of soil microbial communities

**18.Deposit the raw sequence reads into a publicly accessible repository (e.g., https://nmdc.cn/) and provide the corresponding BioProject link in the Data Availability section to ensure transparency and facilitate further research.**

**Response:**

We have followed the reviewer's suggestion and deposited all raw sequence reads generated in this study to the Sequence Read Archive (SRA) of NCBI, under accession numbers PRJNA1302308. And the statement now reads in section 2.2.:*"* *All sequence data have been deposited in the NCBI Sequence Read Archive under accession number PRJNA1302308. "*

**Referrence**

[1]Eilers, K.& Debenport, S.*et al.*. Digging deeper to find unique microbial communities: The strong effect of depth on the structure of bacterial and archaeal communities in soil[J]. Soil Biology and Biochemistry. 2012; 50: 58-65. <https://doi.org/10.1016/j.soilbio.2012.03.011>

[2]Xu, J.& Song, F.*et al.*. Effects of Different Straw Return Methods on the Soil Structure, Organic Carbon Content and Maize Yield of Black Soil Farmland[J]. Agronomy. 2024; 14(9): 2011. <https://doi.org/10.3390/agronomy14092011>

[3]Dong, J.& Wang, P.*et al.*. Granulated straw incorporation with rotary tillage increases the content of soil organic carbon fractions and available nutrients and shifts bacterial communities in East China[J]. Frontiers in Plant Science. 2025; 16: 1520760. <https://doi.org/10.3389/fpls.2025.1520760>
